# High Prevalence and Mechanism Associated With Extended Spectrum Beta-Lactamase-Positive Phenotype in *Laribacter hongkongensis*

**DOI:** 10.3389/fmicb.2021.618894

**Published:** 2021-02-09

**Authors:** Jade L. L. Teng, Ruibang Luo, Bone S. F. Tang, Jordan Y. H. Fong, Li Wang, Lilong Jia, Chloe K. S. Wong, Elaine Chan, Amy W. S. Leung, Gilman K. H. Siu, Tsz-Ho Chiu, Ami M. Y. Fung, Alan K. L. Wu, Man-Lung Yeung, Susanna K. P. Lau, Patrick C. Y. Woo

**Affiliations:** ^1^Department of Microbiology, Li Ka Shing Faculty of Medicine, The University of Hong Kong, Hong Kong, China; ^2^Department of Computer Science, The University of Hong Kong, Hong Kong, China; ^3^Department of Pathology, Hong Kong Sanatorium and Hospital, Hong Kong, China; ^4^Department of Health Technology and Informatics, The Hong Kong Polytechnic University, Hong Kong, China; ^5^Department of Clinical Pathology, Pamela Youde Nethersole Eastern Hospital, Hong Kong, China

**Keywords:** ESBL, *Laribacter hongkongensis*, AmpC beta-lactamase, prevalence, mechanism

## Abstract

In this study, we reported the prevalence and mechanism associated with the extended-spectrum beta-lactamase (ESBL)-positive phenotype in *Laribacter hongkongensis* isolated from patients and fish. Using the inhibition zone enhancement test, 20 (95.2%) of the 21 patient strains and 8 (57.1%) of the 14 fish strains were tested ESBL-positive. However, ESBL genes, including SHV, TEM, CTX-M, GES, and PER, were not detected in all of these 28 *L. hongkongensis* isolates. No ESBL gene could be detected in either the complete genome of *L. hongkongensis* HLHK9 or the draft genome of PW3643. PCR and DNA sequencing revealed that all the 35 *L. hongkongensis* isolates (showing both ESBL-positive and ESBL-negative phenotypes) were positive for the *ampC* gene. When the AmpC deletion mutant, HLHK9*ΔampC*, was subject to the zone enhancement test, the difference of zone size between ceftazidime/clavulanate and ceftazidime was less than 5 mm. When boronic acid was added to the antibiotic disks, none of the 28 “ESBL-positive” isolates showed a ≥ 5 mm enhancement of inhibition zone size diameter between ceftazidime/clavulanate and ceftazidime and between cefotaxime/clavulanate and cefotaxime. A high prevalence (80%) of ESBL-positive phenotype is present in *L. hongkongensis*. Overall, our results suggested that the ESBL-positive phenotype in *L. hongkongensis* results from the expression of the intrinsic AmpC beta-lactamase. Confirmatory tests should be performed before issuing laboratory reports for *L. hongkongensis* isolates that are tested ESBL-positive by disk diffusion clavulanate inhibition test.

## Introduction

*Laribacter hongkongensis* is a globally distributed Gram-negative, facultative anaerobic, motile, S-shaped, asaccharolytic, urease-positive bacillus in the *Chromobacteriaceae* family of beta-proteobacteria (Woo et al., 2003, 2004; Ni et al., 2007; Kim et al., 2011; Beilfuss et al., 2015). *L. hongkongensis* causes highly fatal bacteremic sepsis in patients with underlying liver diseases (Yuen et al., 2001; Kim et al., 2011; Tse et al., 2014; Hung et al., 2020). In addition, it is also associated with fish-borne community-acquired gastroenteritis and traveler’s diarrhea (Woo et al., 2004). *L. hongkongensis* has been found in up to 60% of intestines of commonly consumed freshwater fish of the carp family (Woo et al., 2004; Teng et al., 2005). In the last decade, the complete genome of *L. hongkongensis* was sequenced and the mechanisms employed by it to adapt to various environmental stresses were characterized (Woo et al., 2009; Xiong et al., 2014, 2015, 2017). As for its antibiotic resistance genes and mechanisms, we have previously cloned and characterized the *ampC* and *tetA* genes in *L. hongkongensis* (Lau et al., 2005, 2008). Other groups have also reported various antibiotics resistance mechanisms in *L. hongkongensis* isolates from their localities (Feng et al., 2011; Raja and Ghosh, 2014; Wu et al., 2018; Wang et al., 2019).

Extended-spectrum beta-lactamases (ESBLs) are an extremely important group of beta-lactamases which are usually plasmid-mediated. Since its first discovery in 1979 (Sanders and Sanders, 1979), ESBLs have been increasingly found in Gram-negative bacteria such as *Escherichia coli* and *Klebsiella pneumoniae* (Liu et al., 2018; Bezabih et al., 2021). The presence of ESBL confers resistance to many commonly used beta-lactam antibiotics, including the first, second and third generation cephalosporins. Therefore, an increasing prevalence of ESBL producers has become a serious global health burden (Pitout and Laupland, 2008; Harris et al., 2015). Two years ago, a strain of *L. hongkongensis* with ESBL-positive phenotype (index isolate PW3643) was referred to our laboratory. Although we were able to confirm the ESBL-positive phenotype, no known ESBL gene could be amplified from the isolate. In the literature, one group from India reported the detection of ESBL-positive phenotype from *L. hongkongensis* in 2014 (Raja and Ghosh, 2014). In addition, they also claimed to have successfully amplified ESBL genes in their *L. hongkongensis* strains. In 2019, another group from mainland China has also described the presence of ESBL-positive phenotype in *L. hongkongensis* isolated from frogs and freshwater fish (Wang et al., 2019). However, in these two studies, the gene sequences that could have conferred these ESBL-positive phenotypes were not published (Raja and Ghosh, 2014; Wang et al., 2019). In view of these, we sought a better understanding on the ESBL-positive phenotype in *L. hongkongensis* as well as its mechanism, which have important impact in ESBL detection in clinical microbiology laboratories. In this study, we first described the prevalence of ESBL-positive phenotype in *L. hongkongensis* isolates from patients and freshwater fish in Hong Kong. Then, we looked for the gene(s) that confer this ESBL by traditional PCR amplification and sequencing as well as genome sequencing. As all the sequencing results were negative for ESBL genes, we hypothesized that the ESBL-positive phenotype in *L. hongkongensis* is due to the *ampC* gene we previously described (Lau et al., 2005). We previously reported this novel chromosomal class C beta-lactamase in *L. hongkongensis*, representing the first example of AmpC beta-lactamase in the beta-subdivision of proteobacteria (Lau et al., 2005). Phylogenetic analysis revealed that this novel AmpC was only distantly related to other chromosomal- or plasmid-encoded class C beta-lactamases, sharing <50% amino acid sequence identities, but the kinetic properties of this novel AmpC was compatible with those of a class C beta-lactamase (Lau et al., 2005). To test the hypothesis, we deleted the *ampC* gene of a strain of *L. hongkongensis* and studied its change in phenotype. Finally, we examined the use of a boronic acid-based phenotypic test on all the *L. hongkongensis* strains with ESBL-positive phenotype.

## Materials and Methods

### Bacterial Strains

This study was approved by the Institutional Review Board (IRB) of The University of Hong Kong/Hospital Authority Hong Kong West Cluster, Hong Kong East Cluster Research Ethics Committee. The index isolate refers to the first *L. hongkongensis* strain, PW3643, from which we observed the ESBL-positive phenotype. It was isolated from the stool sample of a patient with gastroenteritis in Hong Kong in 2018. In addition to strain PW3643, the present study also included 34 non-duplicated *L. hongkongensis* strains, 20 of which were previously isolated from patients (Woo et al., 2003, 2004) and 14 from grass carps. For the 20 patient strains, one (HLHK1) was isolated from the blood culture and empyema pus of a patient with bacteremic empyema thoracis (Yuen et al., 2001). The other 19 strains (HLHK2 to HLHK20) were recovered from the stool of patients with community-acquired gastroenteritis (Woo et al., 2003, 2004). For the 14 fish strains, they were isolated from grass carps which were purchased during June to August 2018 from retail markets at three different locations in Hong Kong. The identities of all 35 *L. hongkongensis* strains were confirmed by standard conventional biochemical methods, and 16S ribosomal RNA gene sequencing (Lau et al., 2003; Woo et al., 2004).

### Inhibition Zone Enhancement Test for ESBLs

Since no existing CLSI guidelines were available for ESBL testing of *L. hongkongensis*, ESBL phenotypic tests were performed and interpreted according to methods for testing *Enterobacteriaceae* (CLSI, 2019). Briefly, the inhibition zone enhancement test was carried out by inoculating a 0.5 McFarland standard suspension of overnight culture on Mueller-Hinton agar (Bio-Rad, United States) and applying antibiotic disks (BD BBL^TM^ Sensi-DisccpsdummyTM, Benton Dickinson, United States) of cefotaxime (CTX-30 μg) and ceftazidime (CAZ-30 μg) alone and in combination with clavulanic acid (CTX-CLA 30/10 μg or CAZ-CLA 30/10 μg) following the standard procedure (CLSI, 2019). The plates were incubated at 37°C for 18 h. Upon incubation, the diameter of inhibition zone around each disk was measured following the CLSI guideline. An ESBL-positive phenotype was confirmed when a ≥ 5 mm enhancement of the zone of inhibition was observed in CTX-CLA or CAZ-CLA compared with the diameter of the zone of inhibition of the respective antimicrobial disk alone. *K. pneumoniae* ATCC 700603 and *E. coli* ATCC 25922 were used as positive and negative controls, respectively.

The modified CLSI ESBL phenotypic test developed by Poulou’s group was also performed using the same procedures as described in the previous paragraph with one modification, in which 10 μl of boronic acid (30 mg/ml) was added to the antimicrobial disk before they were deposited onto the inoculated Mueller-Hinton agar (Poulou et al., 2014).

### Bacterial RNA Extraction

*L. hongkongensis* cultures grown to mid-log phase were first stabilized with the RNAprotect bacterial reagent (QIAGEN, Germany) followed by isolation of total RNA using RNeasy Mini kit (QIAGEN, Germany) according to the manufacturer’s protocol. The extracted RNA was treated with RNase-free DNase I (Roche, Switzerland) at 37°C for 45 min to degrade the genomic DNA (Xiong et al., 2017).

### PCR Detection of ESBL and AmpC Beta-Lactamase Genes

Bacterial DNA extraction was performed as described previously (Woo et al., 2004). DNA extracts of all *L. hongkongensis* isolates with the ESBL phenotype were subject to PCR and sequencing with conditions as described by Dallenne et al. (2010) for the detection of ESBL genes. They were also subject to PCR for detection of the *ampC* gene, which encodes the AmpC beta-lactamase, following a previously published protocol with newly designed PCR and DNA sequencing primers (5′-CCAGATTCCGGGCATGGC-3′; 5′-CCATCAGGCCAATGCTCGG-3′) (Lau et al., 2005). The reaction was performed in a GeneAmp PCR System 9,700 with the following conditions: 10 min at 95°C, followed by 40 cycles of 95°C for 30 s, 55°C for 1 min, and 72°C for 1 min, with a final extension at 72°C for 10 min. Standard precautions were taken to avoid PCR contamination, and no false-positive result was observed for negative controls.

### Real-Time Quantitative RT-PCR (qRT-PCR)

qRT-PCR was performed using an ABI7900HT Fast Real Time PCR machine (Applied Biosystems, United States) with FastStart DNA Master SYBR Green I Mix reagent kit (Roche), as described by the manufacturer. The mRNA levels of *ampC* in *L. hongkongensis* isolates with and without ESBL phenotype were measured, respectively, by quantitation of cDNA. The calculated threshold cycle (CT) corresponding to the target gene was calculated as 2^(^*^*Ct*^*^*Target*^^–^ *^*Ct*^*^*Reference)*^, normalized to the *rpoB gene* (Xiong et al., 2017). The sequences of the primers used for qRT-PCR are listed in Table 2.

### Genome Sequencing and Assembly

The draft genome sequence of the index isolate PW3643 was determined by high-throughput sequencing using the Illumina Hi-Seq 1500 platform as described previously (Teng et al., 2017). Genomic DNA was extracted from overnight cultures grown on 5% horse blood agar (BD Benton Dickinson, United States) at 37°C using the genomic DNA purification kit (Qiagen, Germany). Extracted DNA was then sequenced by 151 bp paired-end reads with mean library size of 350 bp. *De novo* assembly was performed using SPAdes (Bankevich et al., 2012).

### Genome Annotation and Phylogenetic Analysis of AmpC Gene

To confirm the species identity at the genome level, intergenomic distances [i.e., average nucleotide identity (ANI) values] between HLHK9 (GenBank accession number NC_012559.1), PW3643 (GenBank accession number LZOW00000000), and the type strain HLHK1^*T*^ (GenBank accession number NZ_AUHR01000001.1) were calculated using web services available at http://enve-omics.ce.gatech.edu/ (Goris et al., 2007). Prediction of protein coding regions and automatic functional annotation of the *L. hongkongensis* genome for strain HLHK9 we published previously (Woo et al., 2009) and that of PW3643 was performed using RAST (Rapid Annotations using Subsystem Technology) server version 2.03 (Aziz et al., 2008). Identification of ESBL-encoding genes was performed by blasting the genomes using the ResFinder 3.1 with default parameters (Zankari et al., 2012).

Phylogenetic tree was constructed based on a 123-aa AmpC protein fragment in MEGA X using the maximum likelihood method based on the Jones-Taylor-Thornton model with uniform rates and bootstrap values calculated from 1,000 replicates (Kumar et al., 2018).

### Construction of AmpC Deletion Mutant of *L. hongkongensis* HLHK9

Unmarked, non-polar deletion of *ampC* was constructed by homologous recombination using the suicide plasmid pCVD442 as described previously (Xiong et al., 2014). Bacterial strains and plasmids and the primers used for the deletion mutagenesis are listed in Tables 1, 2, respectively. Briefly, the in-frame deletion arrangement of *ampC* containing its 5’- and 3’-flanking regions was generated using the overlap PCR method and sub-cloned into pCVD442. The resulting plasmid was transferred into HLHK9 by bacterial conjugation from *E. coli* SM10 λ pir. The selection of allelic replacement was performed as described previously (Xiong et al., 2015) and the mutant strain was further confirmed by PCR using primers (LPW31555/31556) specific for the deleted sequence (Table 2). All mutant strains were confirmed by DNA sequencing.

**TABLE 1:** Bacterial strains and plasmids used in this study.

**Strains or plasmids**	**Relative characteristics**	**Source or references**
**Strains**		
*E. coli* SM10(λ pir)	Donor strain for conjugation	Xiong et al. ^14^
HLHK9	Patient isolate, Cef^+^, Sm^+^	Xiong et al. ^15^
HLHK9Δ*ampC*	HLHK9 derivative with *ampC* deletion	This study
**Plasmid**		
pCVD442	Suicide plasmid; *R6K ori mob RP4 bla sacB*	Xiong et al. ^15^

**TABLE 2:** Primers used in this study.

**Primers**	**Sequence (5′–3′)^a^**
**For mutagenesis of *ampC***	
LPW38006 (*ampC* -UF)	GC**TCTAGA**GTCAATCCGGGGATGGCT
LPW38007 (*ampC* -UR)	CAATTCATGATGACGCCTGTTTGTTCCGTATG
LPW38008 (*ampC* -DF)	CGGAACAAACAGGCGTCATCATGAATTGATTG
LPW38009 (*ampC* -DR)	C**GCATGC**TCCGGAAACTGCCTGGCA
LPW31555 (*ampC* -INF)	CCAGATTCCGGGCATGGC
LPW31556 (*ampC* -INR) For qRT-PCR of *ampC* LPW31555 (*ampC* -INF) LPW31556 (*ampC* -INR) LPW21635 (*rpoB*-F) LPW21636 (*rpoB*-R)	CCATCAGGCCAATGCTCGG CCAGATTCCGGG CATGGC CCATCAGGCCAATGCTCGG GTGCTGTTCGTCAATGAG TAGGTCGTAGGATTCTTCG

* ^*a*^Restriction sites in the primer sequences appear in bold.*

### Antimicrobial Susceptibility Testing of AmpC Deletion Mutant and Wild Type *L. hongkongensis* HLHK9

*In vitro* antimicrobial susceptibility testing was performed in triplicate using the broth microdilution method according to CLSI guidelines (CLSI, 2019). Freshly prepared in-house 96-well microtiter panels were used to test the antimicrobial agents at their respective concentration ranges [ampicillin: 0.05–128 mg/L; amoxicillin/clavulanate: 0.03125–128 mg/L (at 2:1 ratio as recommended by CLSI); ceftazidime: 1–256 mg/L; ceftriaxone: 0.25–512 mg/L; cefuroxime: 2–128 mg/L]. The minimum inhibitory concentration of antimicrobial agents was defined as the lowest concentration that inhibited visible growth of the microorganism and results were interpreted as susceptible, intermediate or resistant according to the CLSI MIC breakpoints recommended for *Enterobacteriaceae* (CLSI, 2019). Control strains *Staphylococcus aureus* ATCC25923 and ATCC 29213, and *E. coli* ATCC 25922 were included in each run.

### Data Availability

The genome sequence of *L. hongkongensis* PW3643 assembled by Illumina reads have been deposited in GenBank under accession number LZOW00000000. The nucleotide sequence for the *ampC* gene of *L. hongkongensis* isolates have been lodged within the GenBank sequence database under accession numbers MT640029- MT640042.

## Results

### Identification of *L. hongkongensis*

All suspected *L. hongkongensis* isolates exhibited the same phenotypic properties and biochemical profiles: All appeared as lactose- negative colonies on cefoperazone MacConkey agar (a selective agar medium for isolation of *L. hongkongensis*), seagull-shapes, motile, Gram-negative, bacteria positive for catalase, cytochrome oxidase, urease and arginine dihydrolase, and negative for sugar oxidation/fermentation. Sequence analysis of the 1,318 bp 16S rRNA gene fragment (nucleotide positions 18–1,335 corresponding to the 16S rRNA gene sequence of HLHK1^*T*^) among these isolates demonstrated ≥ 99% nucleotide identities to that of *L. hongkongensis* HLHK1^*T*^. *In silico* genome-to-genome comparisons showed that ANI values between the type strain HLHK1^*T*^ and PW3643 was 97.8%, while between the type strain HLHK1^*T*^ and HLHK9 was 98.9%, which fall in ANI threshold range (95–96%) for species demarcation.

### Inhibition Zone Enhancement Test for ESBLs

**FIGURE 1:**
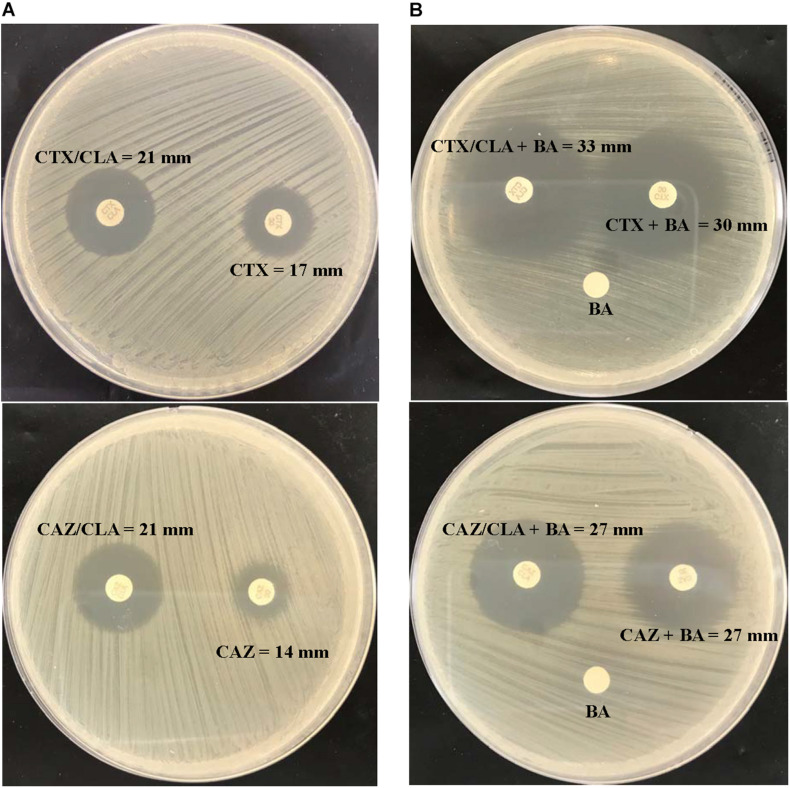
CLSI ESBL confirmatory test of the index isolate PW3643. Results of **(A)** the CLSI ESBL disk diffusion test and **(B)** its modification using antibiotic disks containing boronic acid for the index isolate PW3643. CTX, cefotaxime; CTX/CLA, cefotaxime and clavulanic acid; CAZ, ceftazidime; CAZ/CLA, ceftazidime and clavulanic acid; BA, boronic acid.

**FIGURE 2:**
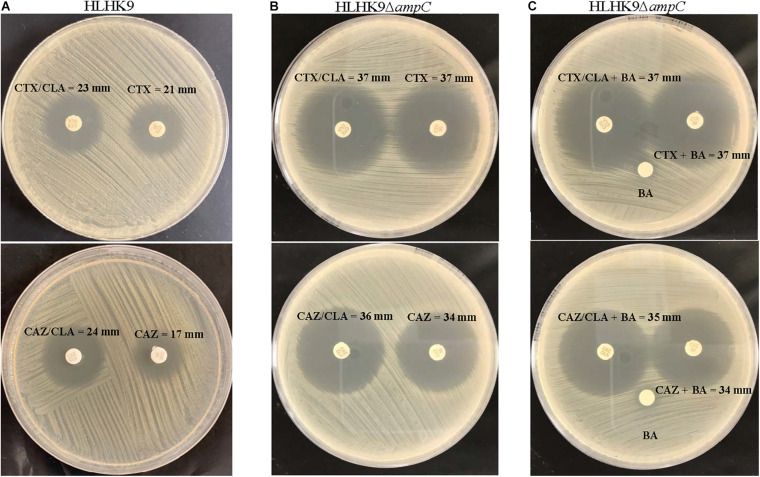
CLSI ESBL confirmatory test of wild type HLHK9 and AmpC deletion mutant HLHK9*ΔampC*. Results of the CLSI ESBL disk diffusion test for **(A)** wild type HLHK9 and **(B)** HLHK9*ΔampC*, and **(C)** its modification using antibiotic disks containing boronic acid for HLHK9*ΔampC*. CTX, cefotaxime; CTX/CLA, cefotaxime and clavulanic acid; CAZ, ceftazidime; CAZ/CLA, ceftazidime and clavulanic acid; BA, boronic acid.

Using the inhibition zone enhancement test, 20 (95.2%) of the 21 *L. hongkongensis* patient strains, including the index isolate, were tested ESBL-positive (Figures 1A,2A and Table 3). One patient isolate (HLHK1^*T*^) was negative for the ESBL-positive phenotype (Table 3). For these 20 patient isolates with ESBL-positive phenotype, 10 (50%; HLHK7, HLHK8, HLHK10-HLHK12, HLHK14, and HLHK16-HLHK19) had a ≥ 5 mm enhancement of inhibition zone around both CTX-CLA and CAZ-CLA when compared with the inhibition zone of the respective antimicrobial disk alone (CTX or CAZ). The other 10 isolates (50%; PW3643, HLHK2-HLHK6, HLHK9, HLHK13, HLHK15, and HLHK20) had a ≥ 5 mm enhancement of inhibition zone around CAZ-CLA but not CTX-CLA when compared with the inhibition zone diameter of the respective antimicrobial disk alone (Table 3). As for the 14 *L. hongkongensis* fish isolates, 8 (57.1%) were tested ESBL-positive (Table 3). Six (75%; F2, F4, F6, F10, F12, and F13) of them had a ≥ 5 mm enhancement of inhibition zone around both CTX-CLA and CAZ-CLA, while the remaining 2 (25%; F3 and F14) had a ≥ 5 mm enhancement of inhibition zone around CAZ-CLA but not CTX-CLA (Table 3).

**TABLE 3:** Results of CLSI ESBL phenotypic confirmatory test with and without the addition of boronic acid.

***L. hongkongensis* isolate**	**PCR detection of the *ampC* gene**	**Without boronic acid (inhibitory zone size in mm)**							**With boronic acid (inhibitory zone size in mm)**	
		**CTX**	**CTX/CLA**	**≥5 mm difference between CTX and CTX/CLA**	**CAZ**	**CAZ CLA**	**≥5 mm difference between CAZ and CAZ/CLA**	**ESBL phenotype**	**CTX + BA**	**CAZ + BA**
**Human isolates (*n* = 21)**										
PW3643	+	17	21	×	14	21	✓	**+**	30	27
HLHK1(Type strain)	+	25	25	×	29	29	×	−	31	34
HLHK2	+	0	0	×	0	23	✓	**+**	26	28
HLHK3	+	9	9	×	0	25	✓	**+**	30	29
HLHK4	+	7	7	×	0	23	✓	**+**	30	28
HLHK5	+	24	24	×	20	28	✓	**+**	27	28
HLHK6	+	22	22	×	15	24	✓	**+**	30	30
HLHK7	+	15	24	✓	7	24	✓	**+**	28	30
HLHK8	+	13	25	✓	0	25	✓	**+**	25	26
HLHK9	+	21	23	×	17	24	✓	**+**	33	30
HLHK10	+	14	25	✓	0	24	✓	**+**	28	27
HLHK11	+	15	25	✓	0	22	✓	**+**	27	25
HLHK12	+	6	20	✓	0	9	✓	**+**	26	28
HLHK13	+	27	27	×	18	25	✓	**+**	30	30
HLHK14	+	22	30	✓	20	26	✓	**+**	28	30
HLHK15	+	21	25	×	17	24	✓	**+**	27	25
HLHK16	+	21	29	✓	18	25	✓	**+**	29	26
HLHK17	+	21	28	✓	14	24	✓	**+**	32	30
HLHK18	+	17	23	✓	15	21	✓	**+**	32	31
HLHK19	+	17	25	✓	0	25	✓	**+**	28	28
HLHK20	+	25	26	×	19	25	✓	**+**	28	26
**Fish isolates (*n* = 14)**										
F1	+	29	32	×	24	27	×	−	33	27
F2	+	16	22	✓	0	21	✓	+	29	26
F3	+	19	23	×	0	22	✓	+	27	27
F4	+	10	21	✓	0	21	✓	+	25	24
F5	+	30	32	×	24	25	×	−	32	28
F6	+	10	20	✓	0	20	✓	+	23	23
F7	+	27	28	×	24	22	×	−	31	28
F8	+	25	25	×	23	22	×	−	29	26
F9	+	27	30	×	22	25	×	−	31	26
F10	+	12	22	✓	0	21	✓	+	23	23
F11	+	27	29	×	25	23	×	−	31	28
F12	+	11	21	✓	0	20	✓	+	24	23
F13	+	11	22	✓	0	20	✓	+	25	25
F14	+	24	25	×	18	24	✓	+	30	27

### PCR Detection of ESBL Genes

Detection of ESBL genes, including SHV, TEM, CTX-M, GES, and PER types, by PCR was negative in all the 28 (20 from patients and 8 from fish) *L. hongkongensis* isolates with ESBL-positive phenotypes.

### Genome Analysis of *L. hongkongensis* HLHK9 and Index Isolate PW3643

In both the complete genome of *L. hongkongensis* HLHK9 and the draft genome of the index isolate PW3643, no known ESBL gene was observed. On the other hand, a putative *ampC* gene encoding an AmpC beta-lactamase was identified in both genomes.

### PCR Detection and Sequencing of AmpC Beta-Lactamase in *L. hongkongensis* Isolates

**FIGURE 3:**
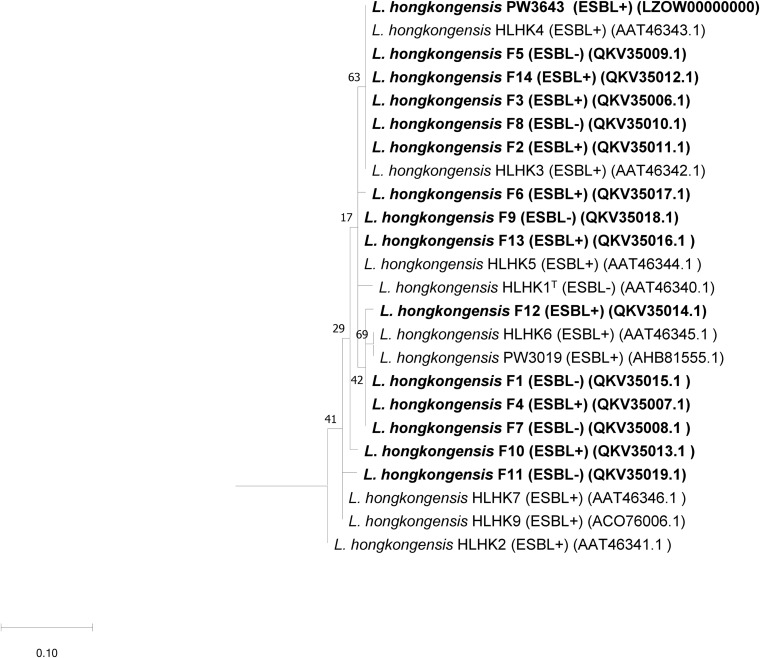
Phylogenetic analysis of the AmpC of “ESBL-positive” and “ESBL-negative” *L. hongkongensis* isolates detected in the present study and their relationship with AmpC sequences of other *L. hongkongensis* isolates. The tree was constructed using the maximum likelihood method and *Pseudomonas oleovorans* (LR130779.1) as the root. Numbers at nodes indicated level of bootstrap support calculated from 1,000 replicates and the scale bar indicates the number of amino acid substitutions per site. Sequences determined in the present study are shown in bold.

**FIGURE 4:**
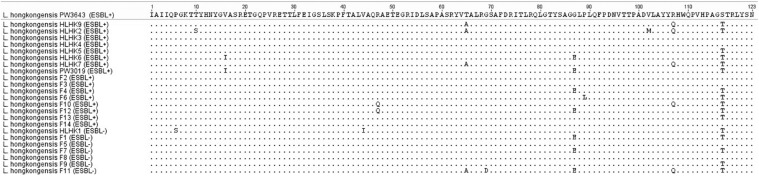
Multiple alignment of amino acid sequences of the AmpC protein from *L. hongkongensis* with different ESBL phenotypes.

**FIGURE 5:**
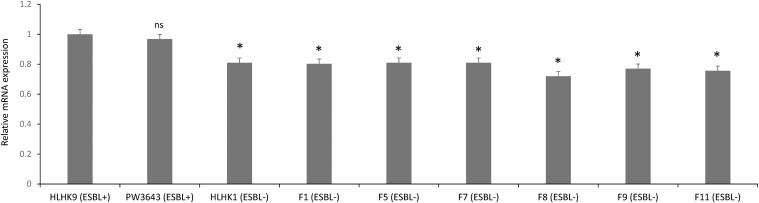
The *ampC* expression in selected *L. hongkongensis* strains. Expression of the *ampC* gene was determined by qRT-PCR in representative strains with different ESBL phenotypes. Error bars represent means ± SEM of three independent experiments. Student’s *t*-test was performed to determine the difference between the *ampC* expression of the selected strains and that of HLHK9. An asterisk indicates a significant difference (ns, not significant; *p < 0.05).

We have confirmed the presence of the *ampC* gene in all *L. hongkongensis* isolates, including the patient isolates HLHK1–HLHK20 (Lau et al., 2005), the index isolate PW3643, and all the fish isolates (8 ESBL-positive and 6 ESBL-negative isolates) (Table 3). Sequence analysis showed high similarities between the patient and fish isolates and they shared 95–100% amino acid identities among each other. The topology of the phylogenetic tree based on the amino acid sequences of AmpC did not reveal any distinct cluster of *L. hongkongensis* isolates with ESBL-positive phenotype (Figure 3). Multiple alignment of the AmpC amino acid sequences also did not reveal any specific mutation associated with each ESBL phenotype (Figure 4). qRT-PCR showed that expression levels of *ampC* in *L. hongkongensis* isolates with ESBL-negative phenotype were significantly lower than those with ESBL-negative phenotype (Figure 5).

### Antimicrobial Susceptibility and ESBL Phenotype of AmpC Deletion Mutant of *L. hongkongensis* HLHK9

To examine the possible role of AmpC in causing the ESBL-positive phenotype in *L. hongkongenesis*, an AmpC deletion mutant, HLHK9*ΔampC*, was generated and subject to the zone enhancement test in parallel with wild type HLHK9 (Figure 2). The *in vitro* activities of eight beta-lactams or beta-lactam/beta-lactamase inhibitor combinations against the AmpC deletion mutant and wild type HLHK9 were also tested. Results showed that the AmpC deletion mutant, HLHK9*ΔampC*, exhibited significant reduction in resistance to ampicillin (MIC from 64 to 1 mg/L), amoxicillin/clavulanate (MIC from 2 to 0.0625 mg/L), ceftazidime (MIC from 128 to 32 mg/L), ceftriaxone (MIC from 128 to 16 mg/L), and cefuroxime (MIC from 32 to 4 mg/L) when compared to the wild type HLHK9. No reduction in resistance was observed on cefepime, imipenem, and cefoperazone/sulbactam, to which the wild type HLHK9 was susceptible. The difference of zone size between CAZ/CLA and CAZ was reduced to less than 5 mm (Figure 2), suggesting that the ESBL-positive phenotype in *L. hongkongnesis* was due to the AmpC beta-lactamase.

### Effect of AmpC Beta-Lactamase on Phenotypic Testing of ESBL

Given that boronic acid has been shown to be an effective inhibitor for AmpC beta-lactamase (Song et al., 2007b; Poulou et al., 2014), a modified CLSI phenotypic confirmatory test based on boronic acid was employed to study the effect of AmpC on the ESBL-positive phenotypes in *L. hongkongensis*. Results showed that upon the addition of boronic acid, all the 28 isolates demonstrated a significant increase in susceptibility to CAZ or CTX, with 15 of them demonstrated an increase in inhibition zone diameter from 0 to > 20 mm (Table 3). Paper disk impregnated with boronic acid only did not result in growth inhibition (Figure 1B).

## Discussion

A high prevalence (80%) of ESBL-positive phenotype is present in *L. hongkongensis* isolated from both patients and fish. ESBL genes are most prevalent in bacteria from the gastrointestinal tract, such as *E. coli* and *K. pneumoniae*. In grass carps, bighead carps and mud carps, *L. hongkongensis* is mainly isolated from their guts (Teng et al., 2005). As for human, *L. hongkongensis* is most commonly associated with freshwater fish borne community-acquired gastroenteritis (Woo et al., 2004). In patients with underlying liver diseases, it translocates through the intestinal wall to the blood stream, causing highly fatal bacteremic sepsis (Yuen et al., 2001; Kim et al., 2011; Hung et al., 2020). In patients undergoing peritoneal dialysis, it can also lead to peritonitis presumably through the same route of infection (Woo et al., 2016). When we first received the index *L. hongkongensis* isolate PW3643 with ESBL-positive phenotype, we thought it might have acquired a plasmid with an ESBL gene from *E. coli*, *K. pneumoniae*, or other members of the *Enterobacteriaceae* family, as we have previously shown that shuttle plasmids that were able to replicate efficiently and propagate in both *E. coli* and *L. hongkongensis* can be easily constructed using plasmids originated from *L. hongkongensis* (Woo et al., 2005). However, PCR detection of the commonly encountered ESBL genes was negative in the index isolate. Therefore, we carried out this study to examine the prevalence and mechanism associated with ESBL-positive phenotype in *L. hongkongensis*. Our results showed that 95.2 and 57.1% of the patient and fish strains, respectively were positive for the ESBL phenotype by the disk diffusion clavulanate inhibition test recommended by CLSI for the detection of ESBL in *Enterobacteriaceae*. However, no known ESBL gene can be amplified from any of these *L. hongkongensis* strains with ESBL-positive phenotypes. We retrieved the complete genome of *L. hongkongensis* HLHK9 which we have manually annotated more than 10 years ago and re-annotated using more sophisticated bioinformatics tools 7 years ago (Woo et al., 2009; Guo et al., 2013). In addition, we also performed whole genome sequencing of the index isolate. In both *L. hongkongensis* genomes, no known ESBL gene(s) were observed. It is indeed intriguing why ESBL genes were claimed to be detected in the previous studies from China to India (Raja and Ghosh, 2014; Wang et al., 2019), however, since no ESBL gene sequences were published, we cannot compare it with our genome sequence. A possibility is that their detected ESBL genes may contain variable genomic differences to known ESBL genes, hence our inability to detect it conventionally, or they may be false-positive PCR results. More sequence data from these two studies would be useful to validate their observations.

Our results suggested that the ESBL-positive phenotype in *L. hongkongensis* results from the expression of the intrinsic AmpC beta-lactamase. As it has been shown previously that the disk diffusion clavulanate inhibition test may give rise to ESBL-positive phenotype, with positive rates ranging from 2.2 to 47.4%, in clinical isolates of *Enterobacteriaceae* that produce chromosomal or plasmid-mediated AmpC beta-lactamase, such as *E. coli*, *Klebsiella* species, and *Enterobacter* species (Tzelepi et al., 2000; Song et al., 2007a; Jeong et al., 2008; Robberts et al., 2009; Poulou et al., 2014), we hypothesized that the ESBL-positive phenotype in *L. hongkongensis* could be due to its *ampC* gene (Lau et al., 2005), which is indeed present in all *L. hongkongensis* isolates tested. The hypothesis was first confirmed by comparing the results of the disk diffusion clavulanate inhibition test for HLHK9 and HLHK9*ΔampC.* For HLHK9, the diameters of the inhibitory zone for CTX, CTX/CLA, CAZ, and CAZ/CLA were 21, 23, 17, and 24 mm, respectively (Figure 2A and Table 3). When the *ampC* gene of HLHK9 was deleted, the corresponding sizes were 37, 37, 34, and 36 mm, respectively (Figure 2B), suggesting that the *ampC* gene of HLHK9 was responsible for its ESBL-positive phenotype. This fact was further confirmed by a boronic acid-based phenotypic test. Since boronic acid is an inhibitor of AmpC beta-lactamase, the inhibitory zones for CTX and CAZ will become larger when boronic acid is added to the corresponding disks. For HLHK9, the diameters of the inhibitory zone for CTX and CAZ with boronic acid were 33 and 30 mm, respectively, as compared to 21 and 17 mm when there was no boronic acid (Figure 2A and Table 3). This phenomenon of inhibitory zones enlargement was observed in all the other 27 human and fish *L. hongkongensis* isolates with the ESBL-positive phenotypes. It is notable that *ampC* gene was in fact present in all *L. hongkongensis* isolates, but only 80% of them showed the ESBL-positive phenotype (Table 3). This may be due to the differential expression of *ampC* in *L. hongkongensis* as the qRT-PCR results showed that the expression of *ampC* in the remaining 20% of *L. hongkongensis* isolates (i.e., ESBL-negative phenotype) were lower than those with the ESBL-positive phenotype (Figure 5). We also noted that *L. hongkongensis* isolates exhibited variability in susceptibility profiles against CAZ and/or CTX, with more isolates resistant to CAZ (Table 3). Given the whole genome sequencing as well as the *ampC* deletion were only performed in a few *L. hongkongensis* isolates, further studies are required to determine whether the variations in resistance profiles of different *L. hongkongensis* isolates is due to the presence of other or unrecognized antimicrobial resistance genes.

Confirmatory tests should be performed before issuing laboratory reports for *L. hongkongensis* isolates that are tested ESBL-positive by disk diffusion clavulanate inhibition test. For severe infections caused by ESBL producing bacteria, the standard treatment is an intravenous carbapenem. Alternatively, piperacillin-tazobactam or the newer beta-lactam-beta-lactamase inhibitor combinations such as ceftazidime-avibactam or ceftolozane-tazobactam can be used if the isolate is susceptible. Cephalosporins alone should be avoided. For *L. hongkongensis*, although there is no study on its optimal treatment, *in vitro* susceptibility testing showed that it has variable susceptibilities to the cephalosporins. For the four cases of severe bacteremia sepsis reported in the literature, the two patients who survived received intravenous cefuroxime and cefotaxime, respectively (Yuen et al., 2001; Kim et al., 2011); whereas the two who succumbed were treated with meropenem (Tse et al., 2014; Hung et al., 2020). Although no statistical conclusions can be drawn, it suggests that cephalosporins can be considered if the isolate is susceptible, which is in line with the absence of ESBL production. As for infection control, recovery of an ESBL producing bacterium from any site requires contact precautions. However, for *L. hongkongensis*, contact precaution is only necessary if the patient has gastroenteritis. Due to the implications on the choice of antibiotics and infection control measures, *L. hongkongensis* with positive ESBL phenotypes that are picked up by disk diffusion clavulanate inhibition test should be at least confirmed with the boronic acid based modified CLSI ESBL phenotypic test. In laboratories with resources in molecular testing, the presence of known ESBL genes can also be confirmed by PCR sequencing.

## Data Availability Statement

The datasets presented in this study can be found in online repositories. The names of the repository/repositories and accession number(s) can be found below: https://www.ncbi.nlm.nih.gov/genbank/, LZOW00000000; https://www.ncbi.nlm.nih.gov/genbank/, MT640029–MT640042.

## Ethics Statement

This study was approved by the Institutional Review Board (IRB) of The University of Hong Kong/Hospital Authority Hong Kong West Cluster, Hong Kong East Cluster Research Ethics Committee. Written informed consent for participation was not required for this study in accordance with the national legislation and the institutional requirements.

## Author Contributions

All authors listed have made a substantial, direct and intellectual contribution to the work, and approved it for publication.

## Conflict of Interest

PW has provided scientific advisory/laboratory services for Gilead Sciences, Incorporated; International Health Management Associates, Incorporated; Merck & Corporation, Incorporated and Pfizer, Incorporated. The remaining authors declare that the research was conducted in the absence of any commercial or financial relationships that could be construed as a potential conflict of interest.

## References

[B1] Aziz R. K. Bartels D. Best A. A. DeJongh M. Disz T. Edwards R. A. (2008). The RAST Server: rapid annotations using subsystems technology. *BMC Genomics* 9:75. 10.1186/1471-2164-9-75 18261238 PMC2265698

[B2] Bankevich A. Nurk S. Antipov D. Gurevich A. A. Dvorkin M. Kulikov A. S. (2012). SPAdes: a new genome assembly algorithm and its applications to single-cell sequencing. *J. Comput. Biol.* 19 455–477. 10.1089/cmb.2012.0021 22506599 PMC3342519

[B3] Beilfuss H. A. Quig D. Block M. A. Schreckenberger P. C. (2015). Definitive Identification of Laribacter hongkongensis Acquired in the United States. *J. Clin. Microbiol.* 53 2385–2388. 10.1128/JCM.00539-15 25948608 PMC4473238

[B4] Bezabih Y. M. Sabiiti W. Alamneh E. Bezabih A. Peterson G. M. Bezabhe W. M. (2021). The global prevalence and trend of human intestinal carriage of ESBL-producing *Escherichia coli* in the community. *J. Antimicrob. Chemother.* 76 22–29. 10.1093/jac/dkaa399 33305801

[B5] CLSI (2019). *Clinical and Laboratory Standards Institute (CLSI). Performance Standards for Antimicrobial Susceptibility Testing. CLSI supplement M100*, 29 Edn Pennsylvania: CLSI.

[B6] Dallenne C. Da Costa A. Decre D. Favier C. Arlet G. (2010). Development of a set of multiplex PCR assays for the detection of genes encoding important beta-lactamases in *Enterobacteriaceae*. *J. Antimicrob. Chemother.* 65 490–495. 10.1093/jac/dkp498 20071363

[B7] Feng J. L. Yan H. Chowdhury N. Neogi S. B. Yamasaki S. Shi L. (2011). Identification and characterization of integron-associated antibiotic resistant Laribacter hongkongensis isolated from aquatic products in China. *Int. J. Food Microbiol.* 144 337–341. 10.1016/j.ijfoodmicro.2010.10.014 21075469

[B8] Goris J. Konstantinidis K. T. Klappenbach J. A. Coenye T. Vandamme P. Tiedje J. M. (2007). DNA-DNA hybridization values and their relationship to whole-genome sequence similarities. *Int. J. Syst. Evol. Microbiol.* 57 81–91. 10.1099/ijs.0.64483-0 17220447

[B9] Guo F. B. Xiong L. Teng J. L. Yuen K. Y. Lau S. K. Woo P. C. (2013). Re-annotation of protein-coding genes in 10 complete genomes of Neisseriaceae family by combining similarity-based and composition-based methods. *DNA Res.* 20 273–286. 10.1093/dnares/dst009 23571676 PMC3686433

[B10] Harris P. N. Tambyah P. A. Paterson D. L. (2015). beta-lactam and beta-lactamase inhibitor combinations in the treatment of extended-spectrum beta-lactamase producing *Enterobacteriaceae*: time for a reappraisal in the era of few antibiotic options? *Lancet Infect. Dis.* 15 475–485. 10.1016/s1473-3099(14)70950-8 25716293

[B11] Hung D. L. L. Teng J. L. L. Fong J. Y. H. Wang Q. Chen Z. X. Fung A. M. Y. (2020). Severe underlying liver diseases and high mortality associated with Laribacter hongkongensis bacteremia. *Diagn. Microbiol. Infect. Dis.* 96:114948. 10.1016/j.diagmicrobio.2019.114948 31787408

[B12] Jeong S. H. Song W. Park M. J. Kim J. S. Kim H. S. Bae I. K. (2008). Boronic acid disk tests for identification of extended-spectrum beta-lactamase production in clinical isolates of *Enterobacteriaceae* producing chromosomal AmpC beta-lactamases. *Int. J. Antimicrob. Agents* 31 467–471. 10.1016/j.ijantimicag.2007.12.014 18337065

[B13] Kim D. S. Wi Y. M. Choi J. Y. Peck K. R. Song J. H. Ko K. S. (2011). Bacteremia caused by Laribacter hongkongensis misidentified as Acinetobacter lwoffii: report of the first case in Korea. *J Korean Med. Sci.* 26 679–681. 10.3346/jkms.2011.26.5.679 21532861 PMC3082122

[B14] Kumar S. Stecher G. Li M. Knyaz C. Tamura K. (2018). MEGA X: Molecular Evolutionary Genetics Analysis across Computing Platforms. *Mol. Biol. Evol.* 35 1547–1549. 10.1093/molbev/msy096 29722887 PMC5967553

[B15] Lau S. K. P. Ho P. L. Li M. W. S. Tsoi H. W. Yung R. W. H. Woo P. C. Y. (2005). Cloning and characterization of a chromosomal class C beta-lactamase and its regulatory gene in Laribacter hongkongensis. *Antimicrob. Agents Chemother.* 49 1957–1964. 10.1128/Aac.49.5.1957-1964.2005 15855519 PMC1087626

[B16] Lau S. K. P. Wong G. K. M. Li M. W. S. Woo P. C. Y. Yuen K. Y. (2008). Distribution and molecular characterization of tetracycline resistance in Laribacter hongkongensis. *J. Antimicrob. Chemother.* 61 488–497. 10.1093/jac/dkm539 18227089

[B17] Lau S. K. Woo P. C. Hui W. T. Li M. W. Teng J. L. Que T. L. (2003). Use of cefoperazone MacConkey agar for selective isolation of Laribacter hongkongensis. *J. Clin. Microbiol.* 41 4839–4841. 10.1128/jcm.41.10.4839-4841.2003 14532237 PMC254358

[B18] Liu X. Wang Y. Cui L. Li Y. Xue F. Liu J. (2018). A retrospective study on mcr-1 in clinical *Escherichia coli* and *Klebsiella pneumoniae* isolates in China from 2007 to 2016. *J. Antimicrob. Chemother.* 73 1786–1790. 29912359 10.1093/jac/dky092

[B19] Ni X. P. Ren S. H. Sun J. R. Xiang H. Q. Gao Y. Kong Q. X. (2007). Laribacter hongkongensis isolated from a patient with community-acquired gastroenteritis in Hangzhou City. *J. Clin. Microbiol.* 45 255–256. 10.1128/JCM.01400-06 17021061 PMC1828964

[B20] Pitout J. D. Laupland K. B. (2008). Extended-spectrum beta-lactamase-producing *Enterobacteriaceae*: an emerging public-health concern. *Lancet Infect. Dis.* 8 159–166. 10.1016/s1473-3099(08)70041-0 18291338

[B21] Poulou A. Grivakou E. Vrioni G. Koumaki V. Pittaras T. Pournaras S. (2014). Modified CLSI extended-spectrum beta-lactamase (ESBL) confirmatory test for phenotypic detection of ESBLs among *Enterobacteriaceae* producing various beta-lactamases. *J. Clin. Microbiol.* 52 1483–1489. 10.1128/JCM.03361-13 24574283 PMC3993656

[B22] Raja K. M. Ghosh A. R. (2014). Molecular Insight of Putative Pathogenicity Markers with ESBL Genes and Lipopolysaccharide in Laribacter hongkongensis. *Appl. Biochem. Biotechnol.* 174 1935–1944. 10.1007/s12010-014-1163-0 25154369

[B23] Robberts F. J. Kohner P. C. Patel R. (2009). Unreliable extended-spectrum beta-lactamase detection in the presence of plasmid-mediated AmpC in *Escherichia coli* clinical isolates. *J. Clin. Microbiol.* 47 358–361. 10.1128/JCM.01687-08 19109470 PMC2643682

[B24] Sanders C. C. Sanders W. E. Jr. (1979). Emergence of resistance to cefamandole: possible role of cefoxitin-inducible beta-lactamases. *Antimicrob. Agents Chemother.* 15 792–797. 10.1128/aac.15.6.792 314270 PMC352760

[B25] Song W. Bae I. L. Lee Y. N. Lee C. H. Lee S. H. Jeong S. H. (2007a). Detection of extended-spectrum beta-lactamases by using boronic acid as an AmpC beta-lactamase inhibitor in clinical isolates of Klebsiella spp. and *Escherichia coli*. *J. Clin. Microbiol.* 45 1180–1184. 10.1128/Jcm.02322-06 17301276 PMC1865824

[B26] Song W. Jeong S. H. Kim J. S. Kim H. S. Shin D. H. Roh K. H. (2007b). Use of boronic acid disk methods to detect the combined expression of plasmid-mediated AmpC beta-lactamases and extended-spectrum beta-lactamases in clinical isolates of Klebsiella spp., *Salmonella* spp., and *Proteus mirabilis*. *Diagn. Microbiol. Infect. Dis.* 57 315–318. 10.1016/j.diagmicrobio.2006.08.023 17174510

[B27] Teng J. L. L. Yeung M. L. Chan E. Jia L. Lin C. H. Huang Y. (2017). PacBio But Not Illumina Technology Can Achieve Fast, Accurate and Complete Closure of the High GC, Complex Burkholderia pseudomallei Two-Chromosome Genome. *Front. Microbiol.* 8:1448. 10.3389/fmicb.2017.01448 28824579 PMC5539568

[B28] Teng J. L. Woo P. C. Ma S. S. Sit T. H. Ng L. T. Hui W. T. (2005). Ecoepidemiology of Laribacter hongkongensis, a novel bacterium associated with gastroenteritis. *J. Clin. Microbiol.* 43 919–922. 10.1128/JCM.43.2.919-922.2005 15695706 PMC548085

[B29] Tse C. W. Curreem S. O. Cheung I. Tang B. S. Leung K. W. Lau S. K. (2014). A novel MLST sequence type discovered in the first fatal case of Laribacter hongkongensis bacteremia clusters with the sequence types of other human isolates. *Emerg. Microbes Infect.* 3:e41. 10.1038/emi.2014.39 26038743 PMC4078790

[B30] Tzelepi E. Giakkoupi P. Sofianou D. Loukova V. Kemeroglou A. Tsakris A. (2000). Detection of extended-spectrum beta-lactamases in clinical isolates of *Enterobacter cloacae* and *Enterobacter* aerogenes. *J. Clin. Microbiol.* 38 542–546. 10.1128/jcm.38.2.542-546.2000 10655342 PMC86144

[B31] Wang L. Fu L. W. Liu Z. H. Guo H. J. Wang L. Feng M. (2019). Comparative Analysis of Antimicrobial Resistance, Integrons, and Virulence Genes Among Extended-Spectrum beta-Lactamase-Positive Laribacter hongkongensis from Edible Frogs and Freshwater Fish. *Microb. Drug Resist.* 25 855–864. 10.1089/mdr.2018.0366 30767721

[B32] Woo P. C. Kuhnert P. Burnens A. P. Teng J. L. Lau S. K. Que T. L. (2003). Laribacter hongkongensis: a potential cause of infectious diarrhea. *Diagn. Microbiol. Infect. Dis.* 47 551–556. 10.1016/s0732-8893(03)00161-5 14711474

[B33] Woo P. C. Lau S. K. Teng J. L. Que T. L. Yung R. W. Luk W. K. (2004). Association of Laribacter hongkongensis in community-acquired gastroenteritis with travel and eating fish: a multicentre case-control study. *Lancet* 363 1941–1947. 10.1016/S0140-6736(04)16407-6 15194253

[B34] Woo P. C. Lau S. K. Tse H. Teng J. L. Curreem S. O. Tsang A. K. (2009). The complete genome and proteome of Laribacter hongkongensis reveal potential mechanisms for adaptations to different temperatures and habitats. *PLoS Genet.* 5:e1000416. 10.1371/journal.pgen.1000416 19283063 PMC2652115

[B35] Woo P. C. Ma S. S. Teng J. L. Li M. W. Kao R. Y. Lau S. K. (2005). Construction of an inducible expression shuttle vector for Laribacter hongkongensis, a novel bacterium associated with gastroenteritis. *FEMS Microbiol. Lett.* 252 57–65. 10.1016/j.femsle.2005.08.026 16165318

[B36] Woo P. C. Poon R. W. Foo C. H. To K. K. Lau S. K. (2016). First Report of Laribacter hongkongensis Peritonitis in Continuous Ambulatory Peritoneal Dialysis. *Perit. Dial. Int.* 36 105–107. 10.3747/pdi.2014.00270 26838992 PMC4737574

[B37] Wu H. K. Chen J. H. Yang L. Li A. R. Su D. H. Lin Y. P. (2018). Emergence and genomic analysis of MDR Laribacter hongkongensis strain HLGZ1 from Guangzhou, China. *J. Antimicrob. Chemother.* 73 643–647. 10.1093/jac/dkx470 29244123

[B38] Xiong L. Teng J. L. Watt R. M. Kan B. Lau S. K. Woo P. C. (2014). Arginine deiminase pathway is far more important than urease for acid resistance and intracellular survival in Laribacter hongkongensis: a possible result of arc gene cassette duplication. *BMC Microbiol.* 14:42. 10.1186/1471-2180-14-42 24533585 PMC3936950

[B39] Xiong L. Teng J. L. Watt R. M. Liu C. Lau S. K. Woo P. C. (2015). Molecular characterization of arginine deiminase pathway in Laribacter hongkongensis and unique regulation of arginine catabolism and anabolism by multiple environmental stresses. *Environ. Microbiol.* 17 4469–4483. 10.1111/1462-2920.12897 25950829

[B40] Xiong L. Yang Y. Ye Y. N. Teng J. L. Chan E. Watt R. M. (2017). Laribacter hongkongensis anaerobic adaptation mediated by arginine metabolism is controlled by the cooperation of FNR and ArgR. *Environ. Microbiol.* 19 1266–1280. 10.1111/1462-2920.13657 28028888

[B41] Yuen K. Y. Woo P. C. Teng J. L. Leung K. W. Wong M. K. Lau S. K. (2001). Laribacter hongkongensis gen. nov., sp. nov., a novel gram-negative bacterium isolated from a cirrhotic patient with bacteremia and empyema. *J. Clin. Microbiol.* 39 4227–4232. 10.1128/JCM.39.12.4227-4232.2001 11724825 PMC88529

[B42] Zankari E. Hasman H. Cosentino S. Vestergaard M. Rasmussen S. Lund O. (2012). Identification of acquired antimicrobial resistance genes. *J. Antimicrob. Chemother.* 67 2640–2644. 10.1093/jac/dks261 22782487 PMC3468078

